# Transcriptome Analysis Reveals Circadian Rhythmic Regulation of Lipid Metabolism and Immune Function in Chicken Livers

**DOI:** 10.3390/ani15223241

**Published:** 2025-11-08

**Authors:** Jiahua Li, Jie Dong, Minjie Huang, Yuting Jin, Xiaodong Tan, Deqian Wang

**Affiliations:** 1 Institute of Animal Husbandry and Veterinary Science, Zhejiang Academy of Agricultural Sciences, Hangzhou 310021, China; xiahua0526@163.com (J.L.); dongj@zaas.ac.cn (J.D.); huangmj@zaas.ac.cn (M.H.); jinyvting1@163.com (Y.J.); 2 Key Laboratory of Livestock and Poultry Resources (Poultry) Evaluation and Utilization, Ministry of Agriculture and Rural Affairs, Hangzhou 310021, China

**Keywords:** chicken, hepatic rhythm, lipid metabolism, immune, transcriptome

## Abstract

**Simple Summary:**

In this study, liver samples were collected at 7 time points during one light/dark cycle, and transcriptomic sequencing was used to explore candidate genes and pathways associated with hepatic rhythm. Trend analysis showed that genes such as *FAM21C*, *SRSF4*, *TLR2A*, *MSMO1*, *ELOVL2*, and *HMGCR* exhibited rhythmic variation trends. A total of 845 differentially expressed genes (e.g., *MSMO1*, *FAM21C*) were detected between light/dark conditions, among which the changes in the activity of lipid metabolism and immune function were the most significant. Weighted gene co-expression network analysis revealed that two modules were strongly associated with the hepatic circadian rhythm. Cosinor analysis showed that 9 lipid-related genes (e.g., *MSMO1*, *HMGCR1*, *ELOVL2*) and 11 immune-related genes (e.g., *FAM21C*, *TLR4*, *TLR2A*) exhibited significant rhythmic expression.

**Abstract:**

Liver rhythm has a significant effect on lipid metabolism and immune function in chickens. However, reports on its underlying mechanisms and key genes are relatively scarce. We collected liver samples at seven time points during one light/dark cycle and investigated the candidate genes and pathways related to hepatic rhythm through transcriptomic sequencing. Trend analysis revealed that the expression of genes in Profile 5 exhibited rhythmic fluctuations, and these genes (e.g., *FAM21C*, *SRSF4*, and *TLR2A*) were enriched in immune function and biological rhythm. The genes (e.g., *MSMO1*, *ELOVL2*, and *HMGCR*) in Profile 2 that were related to lipid metabolism also exhibited a rhythmic trend. A total of 845 differentially expressed genes (e.g., *MSMO1* and *FAM21C*) were detected between light/dark conditions. Lipid metabolism and immune functions showed the most changes between the two conditions. Immune-related processes (e.g., autophagy) were more active in the light phase, while in the dark phase, lipid metabolism (e.g., sterol biosynthesis) was more active. Weighted gene coexpression network analysis revealed that the tan (including *C1QA*, *TLR2A*, and others) and cyan (including *ELOVL2*, *IARS1*, and others) modules were strongly associated with the hepatic circadian rhythm. Cosinor analysis revealed that 9 lipid-related genes (e.g., *MSMO1*, *HMGCR1*, and *ELOVL2*) and 11 immune-related genes (e.g., *FAM21C*, *TLR4*, and *TLR2A*) exhibited significant rhythmic expression. These findings revealed rhythmic changes in hepatic immune and lipid metabolism, providing important insights into the regulation of disease resistance and lipid deposition in chickens.

## 1. Introduction

The biological clock maintains stable circadian rhythmic changes in the physiological functions and behavioral activities of organisms during periodic day–night cycles [1]. The circadian rhythm biological clock can be divided into the master clock and peripheral clocks. In mammals, the master clock, which regulates animal behavior and physiological activities, is located in the suprachiasmatic nucleus (SCN) [2], whereas in birds, the pineal gland, retina, and SCN are independent biological clocks that interact to form a functional unit known as the central clock system [3]. Peripheral clocks are found in organs, tissues, and cells outside the SCN of the hypothalamus. The circadian rhythmicity of the liver is likely the most pronounced; approximately 16% of its transcriptome exhibits circadian rhythmicity, with roles in regulating metabolic processes and maintaining energy homeostasis [4].

Studies have shown that the *CLOCK*, *BMAL1*, *PER1*, *PER2*, *CRY1*, and *CRY2* genes are key factors in the regulation of biological rhythms and play important roles in lipid metabolism and immune function in animals [5]. In mammals, knocking out the *CLOCK* gene causes mice to exhibit circadian rhythm disorders and makes them prone to developing hyperlipidemia and hyperglycemia [6]. The absence of *BMAL1* in mouse livers increases hepatic lipoprotein secretion and reduces cholesterol levels in bile [7]. The circadian rhythm gene *PER1* directly binds to the major hepatic enzymes involved in bile acid synthesis, thereby regulating daily fat absorption and accumulation [8]. Circadian disruption induces the loss or inversion of daily patterns of M1 (proinflammatory) and M2 (anti-inflammatory) macrophages and cytokine levels in spleen and tumor tissues [9]. *BMAL1* controls circadian variations in ConA-induced hepatitis by directly regulating the circadian transcription of Junb and promoting M1 macrophage activation [10]. The absence of the core clock component protein *CRY* leads to constitutive elevation of proinflammatory cytokines in a cell-autonomous manner [11]. Although changes in a variety of traditional clock genes (such as *CLOCK* and *BMAL1*) have important effects, further exploration of related downstream genes is still needed.

Lipid metabolism plays an important role in maintaining lipid homeostasis and regulating immune functions. Zhang et al. used quantitative Real-Time Polymerase Chain Reaction to investigate that immunosuppression inhibits T cells by upregulating steroid anabolism, and significantly suppresses both the natural and specific immune phases of the primary immune response in adipose tissue, as well as the initiation phase of the secondary immune response [12]. In the liver tissue of male chicken embryos, gene expression analysis revealed impaired immune function, which induces cholestatic liver/biliary fibrosis and disorders of lipid and steroid metabolism [13]. Studies have demonstrated that dysregulation of lipid metabolism exerts intricate impacts on the pathogenesis and progression of systemic lupus erythematosus within key effector cells, including T cells, B cells, and innate immune cells [14]. Studies have shown that macrophages are the primary type of immune-infiltrating cells in the subcutaneous adipose tissue of chickens and are associated with immune responses [15].

The liver is an important metabolic organ in the body with crucial roles in maintaining lipid and cholesterol metabolic homeostasis, immune regulation, and other functions [16,17]. The molecular circadian clock can regulate metabolic pathways by rhythmically activating or inhibiting clock-controlled genes involved in metabolism [18]. In lipid metabolism, the hepatic circadian clock is the primary driver that influences the differential and rhythmic expression of fatty acid metabolic pathways [19]. Disruption of the circadian rhythm in the mouse liver leads to abnormalities in bile acid and cholesterol metabolism, resulting in the formation of gallstones, which indicates that the hepatic circadian rhythm is associated with lipid metabolism [20]. In chickens, Wang et al. identified 10 hub genes (*PLK4*, *CLOCK*, *SRD5A3*, etc.) in the liver that are associated with circadian rhythm regulation and lipid metabolism homeostasis [21]. Moreover, in terms of immune regulation, the hepatic circadian rhythm plays an important role in the immune response, and many inflammatory processes in liver diseases are associated with molecular clock dysfunction [10]. REV-ERB links the hepatic circadian clock to the hepatic glucocorticoid receptor, and when the expression of REV-ERB is repressed by more than 80%, it can alleviate the immune response [22]. These circadian rhythm-regulated genes participate in various metabolic processes of the liver (including lipid metabolism and immune metabolism). As the external environment fluctuates rhythmically, they maintain the homeostatic balance of hepatic physiological functions.

In this study, we collected liver samples at seven time points during one light/dark cycle and performed transcriptomic sequencing. Through weighted gene coexpression network analysis (WGCNA) and rhythmic analysis, we identified the regulatory networks and crucial genes influencing hepatic lipid metabolism and immune function. These findings provide important insights into the regulation of disease resistance and lipid deposition in chickens.

## 2. Materials and Methods

### 2.1. Animals

One hundred and five 1-day-old female chicks (Hy-Line Chicken, Body weight range: 28.9–33.3, Jiande Jianke Breeding Co., Ltd., Hangzhou, China) were used in this study. These chickens were randomly divided into seven groups with 3 replicates of 5 individuals per group, no significant difference in body weight was found among each group. All the chickens had access to food and water ad libitum, were reared under 400–700 nm white light (15 ± 0.3 l×) and were housed with a 12:12 h light/dark cycle. The composition and nutrient content of the diets are provided in Table S1. The humidity was controlled at 50–60%. The room temperature was maintained at 33 ± 1 °C for the first week and decreased by 2 to 3 °C per week until it reached 21 °C in the 6th week.

### 2.2. Sample Collection

On day 56, the test chickens were sampled every 4 h. The time points were divided into 7 groups: CT0, CT4, CT8, CT12, CT16, CT20, and CT24 (circadian rhythm time); 1 chicken was randomly slaughtered in each replicate of each group, and the liver tissue samples of the chickens were rapidly collected, quickly placed in liquid nitrogen, and preserved at −80 °C.

### 2.3. Total RNA Extraction, Quality Control, and Sequencing

Total RNA was extracted from liver samples using TRIzol reagent (Sangon Biotech, Shanghai, China), and the purity of total RNA was assessed using a NanoDrop spectrophotometer (Thermo Fisher Scientific, Waltham, MA, USA), while the concentration of total RNA was determined using a Qubit 2.0 fluorometer (Thermo Fisher Scientific, Waltham). After enrichment of polyadenylated eukaryotic mRNA with oligo(dT)-containing magnetic beads, library construction was completed using the Illumina TruSeq RNA Sample Preparation Kit method (Illumina, Inc., San Diego, CA, USA). Qualified and accurately quantified libraries are sequenced using the NovaSeq 6000 platform (Illumina, Inc.).

### 2.4. Data Quality Control, Alignment, and Quantification

Low-quality raw data were discarded using Fastp v0.23.4 [23], and the quality of the clean data was assessed using FastQC v0.12.0 [24]. Filtered reads were aligned to the chicken reference genome GRCg7b using HISAT2 v2.2.1 [25] (--dta), and the results were subjected to file conversion, read sorting, and indexing using SAMtools v1.22.1 [26]. The obtained bam files were used for transcript assembly with the reference annotation GTF file using StringTie v2.2.3 [27] (-A, indicating that only the known transcripts were assembled). Finally, the expression abundance of each gene was calculated using a prepDE.py script (−l 150) and normalized to transcripts per million (TPM) values using an in-house script.

### 2.5. Identification of Differentially Expressed Genes (DEGs) Between Light/Dark Conditions

Only genes (10,439) with an average TPM value ≥ 1 in all the samples were screened for subsequent studies. First, we identified and statistically analyzed the DEGs between different sampling points (CT0 vs. CT4, CT4 vs. CT8, CT8 vs. CT12, CT12 vs. CT16, CT16 vs. CT20, and CT20 vs. CT24). Next, the samples were divided into light/dark groups on the basis of the sampling time, and DEGs between light/dark conditions were also identified. All DEGs were identified using the DESeq2 package v4.2.2 [28] on the basis of a threshold of *p* < 0.05 and a |fold change| > 1.5.

### 2.6. Classification of Gene Expression Trends

To identify candidate genes related to circadian rhythm, a STEM-Short Time series Expression Miner analysis was first conducted. Genes with the same expression trend were classified into one cluster. An adjusted *p* value < 0.05 was defined as the significance threshold.

### 2.7. Weighted Gene Co-Expression Network Analysis

A coexpression network was constructed to identify candidate genes related to light/dark conditions using the WGCNA package v1.72.1 [29]. On the basis of the expression matrix of the 10,439 genes, the soft threshold (β = 14, Figure S1) was determined according to the results of the scale-free distribution (R^2^ > 0.9). Afterward, a step-by-step and dynamic cutting tree was used to construct the coexpression network. The detailed parameters were minModuleSize = 50 and mergeCutHeight = 0.25. A correlation coefficient > 0.6 was set as the threshold for circadian rhythm gene modules. In the circadian rhythm gene module, genes with a |Eigengene-based Connectivity (KME)| > 0.8 were defined as hub genes.

### 2.8. Functional Enrichment Analysis

To clarify the functions of the candidate genes in terms of expression trends, DEGs between light/dark conditions, and candidate gene modules, Kyoto Encyclopedia of Genes and Genomes (KEGG) enrichment analyses were performed using the online tool OmicShare (https://www.omicshare.com/) (accessed on 8 July 2025). A *p* value < 0.05 indicated significant enrichment. Additionally, biochemical reactions and molecules affecting liver rhythms were identified using the Reactome database.

### 2.9. Cosinor Method of Biorhythm Analysis

The rhythmicity of the candidate genes was assessed using the meta2d function (JTK-cycle method) in the MetaCycle package v1.2.0 [30]. Genes whose adjusted *p* value was <0.05 were considered to exhibit circadian expression. In addition, we performed cosinor analysis (https://cosinor.online/appNew/index.php) (accessed on 15 August 2025) to refine and confirm the rhythmic expression of the candidate genes, and a *p* value < 0.05 was defined as the significance threshold.

### 2.10. Validation of Candidate Genes

In this study, nine genes (*FAM21C*, *TLR4*, *TLR2A*, *MSMO1*, *HMGCR1*, *ELOVL2*, *UHRF1*, *CCDC14*, and *RSRP1*) were selected for reverse transcription real-time PCR (RT-qPCR) assays on the basis of expanded samples. The gene-specific primers were designed using Oligo 6.0 software (Table S2). Three biological replicates were established for each sample. The relative expression was calculated using the 2^−△△CT^ method [31].

### 2.11. Statistical Analysis

The expression levels of the candidate genes were analyzed using *t* tests and Pearson correlation analysis, with all the statistical analyses performed using SPSS 25.0 software. *p* < 0.05 was considered to indicate statistical significance.

## 3. Results

### 3.1. Circadian Rhythm of Hepatic Gene Expression

**Figure 1:**
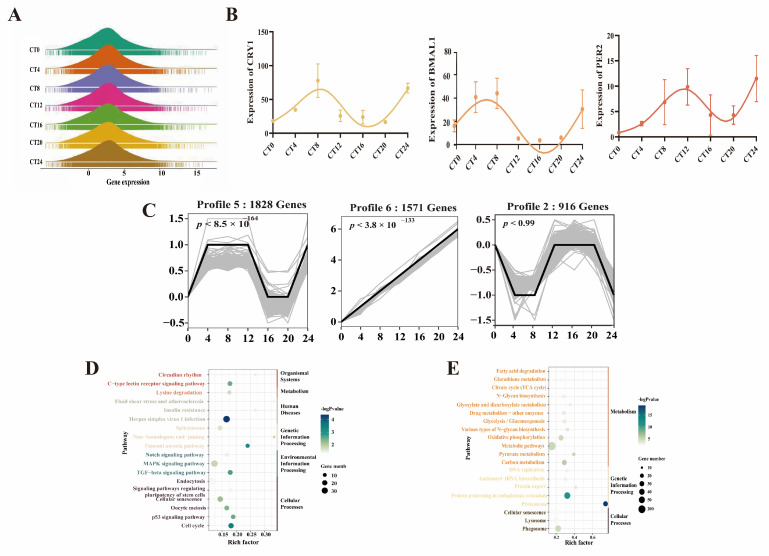
Analysis of hepatic circadian rhythm gene expression. (**A**) Distribution of transcriptional abundance in liver from CT0 to CT24. (**B**) Expression profile of *CRY1*, *BMAL1*, and *PER2* from CT0 to CT24. (**C**) Gene expression trend from CT0 to CT24. (**D**) KEGG enrichment results based on candidate genes in Profile 5. (**E**) KEGG enrichment results based on candidate genes in Profile 6.

Liver samples were collected at seven time points during one light/dark cycle to study the mechanism of hepatic rhythm in chickens. A total of 137.85 Gb of clean data was generated after trimming, and 12,610 genes were detected for transcription (focused on TPM: 0.95~53.77) at one or more time points using the recommended bioinformatic pipeline (Figure 1A). To confirm the biological rhythm model, we quantified the expression of the partial circadian genes *PER2*, *CRY1*, and *BMAL1*. A distinct rhythmic pattern was found for these genes, which ensured a solid model (Figure 1B). In one light/dark cycle, we calculated the expression level in 6 comparisons and found the most DEGs in the comparisons of CT12 vs. CT8 and CT16 vs. CT12 (Table S3). To investigate the transcriptional pattern at 7 time points, 1571 and 1828 genes were assigned to the significant obvious increase (profile 6) and rhythmic fluctuation (profile 5) patterns, respectively, whereas the other genes enriched in the additional 5 patterns showed no obvious trend (Figure 1C and Figure S2). In profile 5, genes related to immune function (e.g., Herpes simplex virus 1 infection, insulin resistance), cell proliferation (e.g., cell cycle, oocyte meiosis), and biological rhythm (e.g., circadian rhythm) clearly changed (Figure 1D). Nutrient (e.g., glucose, amino acid, and fatty acid) and energy (e.g., oxidative phosphorylation, citrate cycle) metabolism were time-dependent on the basis of the genes in profile 6 (Figure 1E), but many metabolic genes (e.g., *MSMO1*, *ELOVL2*, and *HMGCR*) were expressed rhythmically.

### 3.2. Identification of DEGs Between Light/Dark Conditions

**Figure 2:**
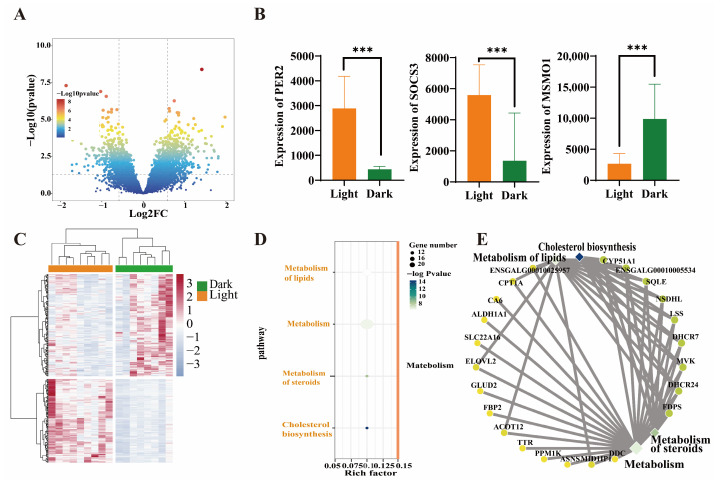
Analysis of differentially expressed genes (DEGs) of chicken liver between light/dark conditions. (**A**) Volcano plot of DEGs between light/dark groups. (**B**) Comparison of gene expression of *PER2*, *SOCS3*, and *MSMO1* between light/dark conditions. *** indicates *p* < 0.001. (**C**) Hierarchical cluster analysis of DEGs between light/dark conditions. (**D**) KEGG enrichment results based on DEGs between light/dark conditions. (**E**) Reactome enrichment results based on DEGs between light/dark conditions.

To identify circadian genes related to hepatic rhythm, we calculated the differences in transcription of genes between light/dark conditions. A total of 845 genes (396 downregulated genes and 449 upregulated genes) were defined as DEGs according to a threshold of a |fold change| > 1.5 and a *p* value < 0.05 (Figure 2A). Genes related to circadian rhythm (*PER2*, *CRY1*, and *HLF*), immune function (e.g., *SOCS3*, *TRAF1*, and *ENSGALG00010003785*), and metabolism (e.g., *MSMO1*, *ELOVL2*, and *HMGCR*) were also significantly enriched (Figure S3). Hierarchical clustering of the expression patterns indicated that the expression profiles of samples from the light/dark groups were divided into distinct clusters (Figure 2C). The KEGG enrichment results revealed that 18 pathways significantly differed between light/dark conditions (Figure 2D). Nutrient metabolism, especially lipid-related metabolism, was clearly enriched. Biological rhythm-related functions were also detected (e.g., circadian rhythm and circadian entrainment). Additionally, the Reactome database was used to identify the biochemical reactions and molecules affecting hepatic rhythm. Lipid metabolism was the most significantly enriched pathway, especially cholesterol and long-chain fatty acid (e.g., linolenic acid) metabolism-related intermediate reactions and production (Figure 2E). In addition, the *DDC* gene, which is highly transcribed in dark environments (Figure S3), plays important roles in the serotonin and melatonin biosynthesis pathways and regulates the biological rhythm of the liver.

### 3.3. WGCNA for Light/Dark

**Figure 3:**
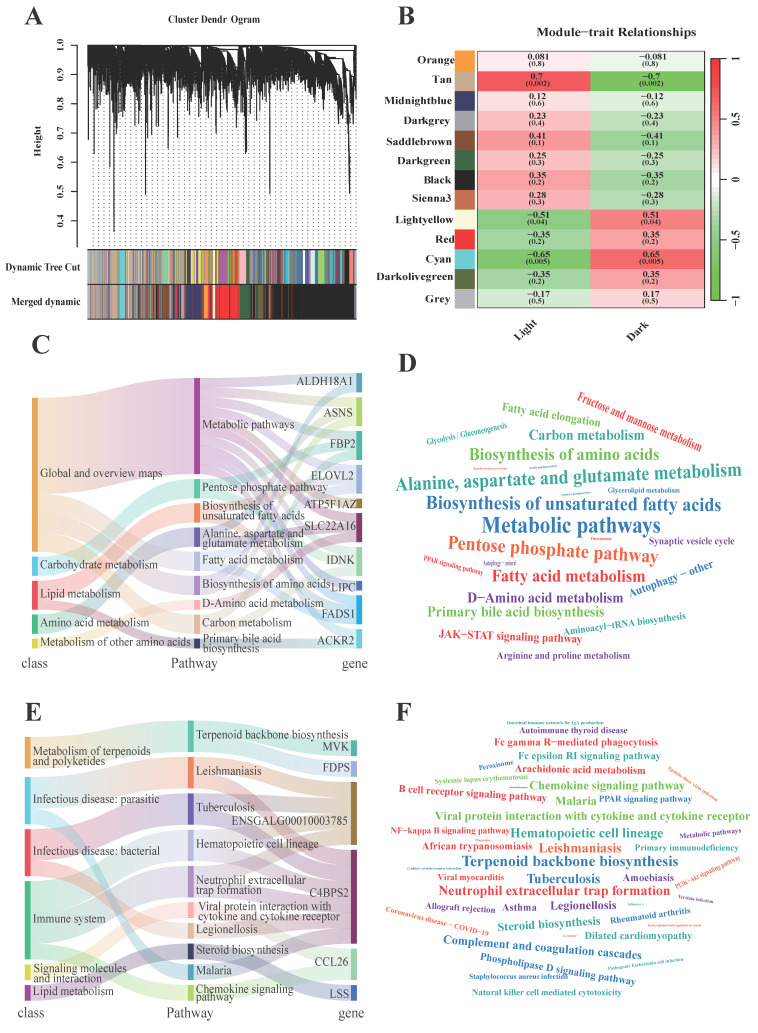
Construction of gene co-expression network related to light/dark conditions. (**A**) Cluster dendrogram of merged gene modules, different colors represent different modules. (**B**) Module-Trait relationship related to light/dark conditions. (**C**) KEGG class and pathways based on the DEGs in cyan module. (**D**) Reactome results based on the DEGs in cyan module. (**E**) KEGG class and pathways based on the DEGs in tan module. (**F**) Reactome results based on the DEGs in cyan module.

Compared with monogenes, gene networks are more likely to participate in the mechanism of hepatic rhythms. We used the WGCNA approach to screen potential gene sets related to functional changes under light/dark conditions. Thirteen gene modules were found after gene merging, and 91~4640 genes were segregated into each module on the basis of the soft thresholding parameter (β = 14, Figure 3A). The cyan and tan modules were significantly correlated with light/dark conditions or related biological functions in the liver (Figure 3B). In the cyan module, 23 DEGs of 278 candidate genes were highly related to metabolic processes involving fatty acids (e.g., linolenic acid and linoleic acid) and amino acids (e.g., lysine) according to the results of the KEGG and Reactome analyses (Figure 3C,D), similar results were also detected based on all genes in cyan module (Figure S4, Table S4). In the tan module, major changes in immune-related functions (e.g., the toll-like receptor signaling pathway, Salmonella infection, and the phagosome) were detected on the basis of 17 DEGs and 310 candidate genes (Figure 3E,F and Figure S5, Table S4). To select hub genes, gene connectivity (>20) and |KME| (>0.8) were calculated, and 82 and 90 hub genes, respectively, were identified in the two potential regulatory networks (Table S4). *BTF3* (46.5) and *ENSGALG00010013482* (51.3) were found to have the greatest degrees in the cyan and tan modules, respectively (Table S5).

### 3.4. Circadian Expression Profile for Candidate Genes

**Figure 4:**
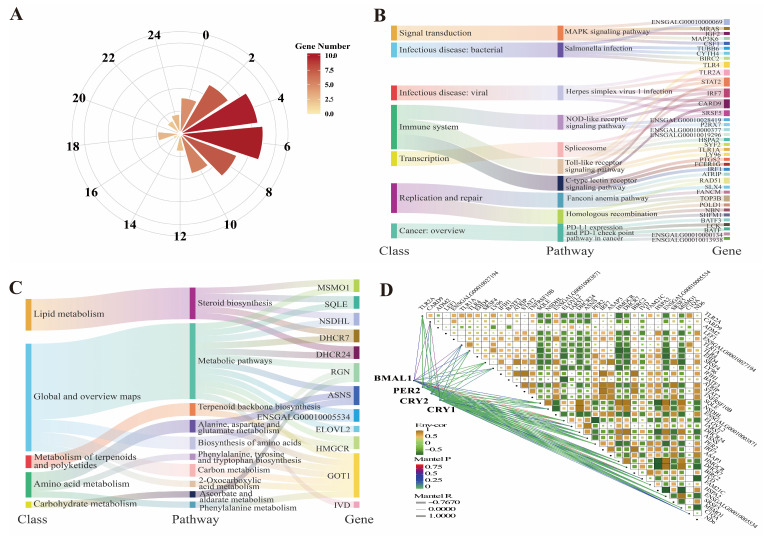
Identification of circadian genes in liver. (**A**) Summary for expressed gene number in light/night phases. (**B**) KEGG enrichment based on the DEGs expressed in light phase. (**C**) KEGG enrichment based on the DEGs expressed in night phase. (**D**) Correlations among circadian genes (*BMAL1*, *PER2*, *CRY1*, *CRY2*) and candidate genes.

To refine the genes associated with hepatic rhythms, a rhythmic expression analysis at seven CTs was performed. A total of 2728 protein-coding genes, including 353 DEGs, were found to have rhythmic significance (Table S6). *PER2*, *CRY1*, and *BMAL1* were shown to have significant rhythmicity, as predicted. We classified all the genes into light/dark conditions because of the phase distribution; a significant gene expression peak occurred during the 11:00~13:00 phase (Figure 4A). In the light, the immune-related functions, including autophagy, Salmonella infection, and apoptosis, showed clear changes in terms of all genes or rhythmic genes (Figure 4B and Figure S6). In the dark, nutrient metabolism, such as fatty acid metabolism, unsaturated fatty acid biosynthesis, the citrate cycle, and amino acid biosynthesis, was more active (Figure 4C and Figure S7). Additionally, 24 immune-related and 12 metabolism-related rhythmic DEGs were significantly associated with known circadian genes (*PER2*, *CRY1*, and *BMAL1*) (Figure 4D). For these genes, we performed cosinor analysis to confirm their rhythmicity and explore their rhythmic characteristics. A total of 9 and 11 candidate genes were correlated with immune and metabolic functions, respectively, including *TLR4*, *TLR2A*, *FAM21C*, *ELOVL2*, *MSMO1*, and *HMGCR* (Table 1). *SRSF4* and *SRSF5* were associated with subsignificant decreases in rhythmic expression (*p* < 0.06). In the correlation heatmap of 20 circadian rhythm-differential genes associated with lipid metabolism and immune function, there are differences in the correlation strength among different genes (Figure S8). Among these genes, the lipid metabolism-related gene *MSMO1* shows a highly significant positive correlation with *DHCR7*, *ENSGALG00010005534*, and *SQLE*, and these four genes also exhibit highly significant positive correlations with one another. The *HMGCR* gene shows a significant positive correlation with the *ENSGALG00010005534* gene, the *SQLE* gene, and the *ELOVL2* gene. Similarly, among the immune function-related genes, the *FAM21C* gene also shows highly significant positive correlations with *CARD9* and *PER2*. The *TLR4* gene exhibits significant positive correlations with *SRSF4* and *LY96*, but a significant negative correlation with *HMGCR*. The *TLR2A* gene shows significant positive correlations with *TLR1A* and *C1QA*.

### 3.5. Validation of Candidate Rhythmic Genes in Liver

**Figure 5:**
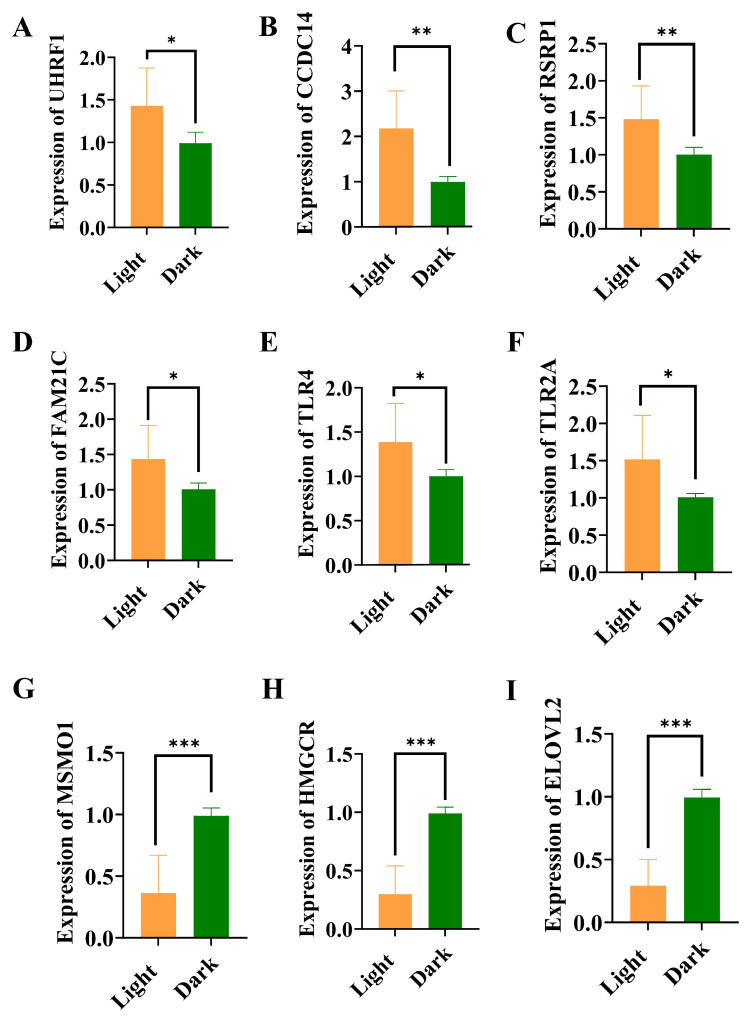
Relative expression levels of candidate DEGs related to hepatic lipid metabolism and immune function. (**A**–**I**) Comparison of gene expression levels between light/dark conditions for *UHRF1*, *CCDC4*, *RSRP1*, *FAM21C*, *TLR4*, *TLR2A*, *MSMO1*, *HMGCR*, and *ELOVL2*. * indicates *p* < 0.05, ** indicates *p* < 0.01, *** indicates *p* < 0.001.

We confirmed the hepatic biological rhythm by quantifying *PER2*, *CRY1*, and *BMAL1*; we then measured the relative expression of randomly selected rhythmic DEGs (*UHRF1*, *CCDC14*, and *RSRP1*) to validate the mRNA sequencing (Figure 5). Similar expression profiles were detected between the sequencing and RT–qPCR results. These three genes were highly transcribed when chickens were subjected to light and exhibited differential expression between light/dark conditions (Figure 5A–C). Additionally, alterations in immune and metabolic functions during one light/dark cycle were verified. The expression of immune-related genes (*FAM21C*, *TLR4*, and *TLR2A*) peaked in the light (Figure 5D–F), whereas the expression of metabolism-related genes (*MSMO1*, *HMGCR1*, and *ELOVL2*) was upregulated in the dark (Figure 5G–I); all these genes were circadian with differential expression between the light/dark conditions, which is consistent with the sequencing results.

## 4. Discussion

The liver plays a central role in lipid biosynthesis and catabolism in poultry, as well as in systemic inflammation and immunity [32]. Disruptions in liver rhythmicity may lead to excessive fat accumulation and liver inflammatory diseases. Therefore, maintaining hepatic rhythmic homeostasis is a critical factor influencing metabolism and health. In this study, liver samples were collected during one light/dark cycle. On the basis of the hepatic gene expression profile, we found that lipid metabolism and immune functions changed rhythmically in the liver. Approximately 21.6% of the hepatic genes exhibited rhythmic expression, including lipid deposition-related genes (*MSMO1*, *ELOVL2*, and *HMGCR*) and immune regulation-related genes (*FAM21C*, *SRSF4*, and *TLR2A*). This study provides preliminary insights into the circadian rhythmic regulatory mechanisms of lipid metabolism and immune regulation in chicken livers.

In chickens, the expression of a large number of genes exhibits regular fluctuations over one circadian cycle, which in turn regulates core physiological processes such as growth, metabolism, and immunity. It is revealed that the content of long-chain polyunsaturated fatty acid and gene expression of *ELOVL5* and *ELOVL2* were all rhythmically changed in liver from pigs [33]. This is consistent with our findings that the *ELOVL2* was found circadian expression in liver, though the animal models were different. It is reported that *HMGCR*, a gene related to cholesterol synthesis, was expressed rhythmically during 12 h light/12 h dark condition [34], our results also confirmed the circadian expression of *HMGCR* in liver. RNA-seq analysis of the airway epithelial response in asthmatic and non-asthmatic horses revealed that the *TLR4* gene was differentially expressed following inhalation challenge, participates in neutrophil chemotaxis, immune and inflammatory responses, and exhibits rhythmic expression patterns [35]. This is highly consistent with the conclusion that the rhythmic expression of *TLR4* regulates immune function.

Among microorganisms, plants, invertebrates and mammals, nearly all biological physiological activities exhibit circadian rhythmic changes [36]. In this study, through trend analysis and rhythmic analysis, Profile 5 showed periodic rhythmic changes. In Profile 5, *FAM21C*, *SRSF4*, and *TLR2A* were significantly enriched in endocytosis, herpes simplex virus 1 infection, and chemical carcinogenesis-reactive oxygen species. In hepatocellular carcinoma (HCC) tissues, high expression of the *FAM21C* gene can promote the malignant progression of HCC, drive the remodeling of the F-actin cytoskeleton, and ultimately increase the invasion and migration of HCC cells [37]. Reducing the expression of *SRSF4* can significantly impair the replication of enterovirus A71 and other enterovirus species [38]. *TLR2A* binds to the corresponding PAMP and triggers a signaling cascade, leading to activation of the transcription factor NF-κB and mitogen-activated protein kinases (MAPKs), which subsequently induce inflammatory cytokines [39]. The genes in Profile 2 also show rhythmic fluctuations. Although these fluctuations are not significant at the overall profile level, some genes (*MSMO1*, *ELOVL2*, and *HMGCR*) do exhibit significant rhythmic expression. Among these genes, *MSMO1* and *SQLE* are significantly enriched in metabolic pathways, fatty acid degradation, and nutrient metabolism pathways [40]. Studies have shown that *MSMO1* significantly increases TG levels and reduces TC levels [41,42]. Increased *MSMO1* expression promotes steroid biosynthesis and influences lipid metabolism processes [43]. *ELOVL2* is involved in the elongation of long-chain fatty acids and is highly expressed in the livers of laying hens [44,45,46]. Liver *HMGCR* expression is positively correlated with serum lipid cholesterol levels [47]. *HMGCR* inhibitors can inhibit cholesterol biosynthesis, accelerate cholesterol metabolism, and thereby regulate lipid metabolism disorders [48,49]. It is revealed that both *HMGCR* and *DHCR7* are involved in hepatic cholesterol metabolism in Shaoxing duck [50]. Additionally, *DHCR7* could downregulate *NSDHL* and led to decreases in bile acid levels [51]. Therefore, lipid metabolism and immune function were the most important functions whose expression changed rhythmically during one light/dark cycle, and genes such as *ELOVL2* and *TLR4* may be the main genes mediating the rhythmic changes in these functions.

As an important environmental zeitgeber, the light/dark cycle strongly influences lipid metabolism and immune homeostasis by regulating biological rhythms [52,53]. Here, DEGs (e.g., *ELOVL2* and *TLR4*) between light/dark conditions were enriched mostly in immune function in the light phase, whereas those involved in lipid metabolism (especially steroid biosynthesis) pathways were more active in the liver in the dark phase. Paredes et al. reported that the expression of lipolytic genes was increased between CT 2:17 h and CT 18:31 h and that the expression of lipogenic genes was increased between CT 15:25 h and 20:06 h (dark phase) in zebrafish [54]. Similar results were reported by Feng et al., who reported that hepatic *FASN* expression significantly increased under dark conditions [55]. Increased hepatic lipid metabolism has been shown in both mice and humans [56]. which is highly consistent with our findings. Additionally, immune functions (e.g., the Toll-like receptor signaling pathway) change significantly between the light/dark cycle and complete dark conditions [57]. Here, we report that immune-related genes (*FAM21C*, *TLR4*, and *TLR2A*) are highly transcribed during the light phase. Similarly to other animals and humans, chickens primarily forage and are active during the daytime. During this period, they may encounter more environmental changes that stimulate immune system activation, and immune-related genes are highly transcribed at this time, enabling them to quickly respond to external pathogens. Continuous illumination enhances the immunity of crabs, and the expression of genes related to immune response varies with the photoperiod [58]. During the night, chickens typically fast and rest, lipid-related genes play a dominant role during this phase, allowing ingested nutrients to be assimilated and stored [59]. Studies have shown that the rate of lipid uptake and metabolism increases during the dark phase [60]. Disruption of natural light/dark cycles, through light at night, impairs innate and adaptive immune responses in nocturnal rodents [61,62].

Furthermore, this study utilized WGCNA and two rhythmic expression pattern analysis methods to identify 9 (e.g., *ELOVL2*, *SQLE*, and *DHCR7*) and 11 (e.g., *C1QA*, *TLR4*, and *CARD9*) rhythmically expressed genes associated with lipid metabolism and immune function, respectively. The 20 circadian rhythm related genes associated with lipid metabolism and immune function exhibit a synergistic effect with the core clock genes (*CLOCK*, *CRY1*, and *CRY2*). The *PER2*, *BMAL1* and *FAM21C* genes belonged to the black module, while *NSDHL*, *IVD*, *SQLE* and *DHCR7* belonged to the red module. Among these, *CARD9* is a crucial component in the regulation of Lyn-mediated Toll-like receptor (*TLR2* and *TLR4*) signaling in dendritic cells [63]. *TLR4* exhibits a strong positive correlation with *PER2,* elevated melatonin levels can induce circadian expression changes in *PER2*, exerting a positive effect on the expression of *TLR4* associated with nighttime sleep [64]. *CRY* may prevent ovarian tissue damage caused by polycystic ovary syndrome through the regulating the expression and function of *HMGB1*, *TLR4*, and NF-κB [65]. In BMAL1 KO mice, Chuanbaipisu increased the expression level of *HMGCR* mRNA and also contributed to elevated cholesterol levels in the liver and serum [66]. In response to the regulation of clock genes (*BMAL1*, *PER2*, and *CRY1*), *ELOVL2*, *TLR4*, and other genes played important role in changes and regulation of lipid and immune function in chicken liver.

## 5. Conclusions

In summary, this study revealed that lipid metabolism (sterol synthesis) and immune function exhibit pronounced circadian oscillations in the liver. We constructed a circadian gene expression profile in chicken liver and characterized 9 lipid-related genes (e.g., *MSMO1*, *HMGCR1*, and *ELOVL2*) and 11 immune-related genes (e.g., *FAM21C*, *TLR4*, and *TLR2A*) exhibited significant rhythmic expression. Under the regulation of circadian clock genes (*PER2*, *BMAL1*, and *CRY1*), these genes may mediate the rhythmic regulation of hepatic immune function and lipid metabolism through sterol and *TLR* pathways. These findings provide insights into the role of biological rhythms in improving animal production and welfare.

## Figures and Tables

**Table 1:** Candidate circadian genes related to hepatic lipid metabolism and immune function.

Gene	*p* Value_DEG	Log2FoldChange	*p* Value_JTK	*p* Value_Cosinor	KEGG
*FAM21C*	4.44 × 10^−4^	6.48 × 10^−1^	9.78 × 10^−5^	0.029	Endocytosis
*SRSF4*	6.06 × 10^−3^	5.95 × 10^−1^	3.70 × 10^−3^	0.050	Herpes simplex virus 1 infection, Spliceosome
*LY96*	1.57 × 10^−3^	6.86 × 10^−1^	6.58 × 10^−3^	0.052	Toll-like receptor signaling pathway, Salmonella infection
*CARD9*	3.20 × 10^−3^	7.78 × 10^−1^	7.76 × 10^−3^	0.025	Herpes simplex virus 1 infection, Spliceosome, NOD-like receptor signaling pathway
*TLR4*	2.05 × 10^−3^	6.61 × 10^−1^	2.53 × 10^−2^	0.023	Toll-like receptor signaling pathway, Salmonella infection
*C1QA*	3.15 × 10^−2^	5.92 × 10^−1^	2.92 × 10^−2^	0.045	Chagas disease, Complement and coagulation cascades
*MSMO1*	1.41 × 10^−6^	−1.88 × 10^0^	5.40 × 10^−3^	0.050	Steroid biosynthesis
*ENSGALG00010005534*	2.00 × 10^−12^	−1.88 × 10^0^	1.54 × 10^−2^	0.025	Metabolic pathways, Terpenoid backbone biosynthesis
*HMGCR*	4.61 × 10^−12^	−1.98 × 10^0^	1.54 × 10^−2^	0.007	Metabolic pathways, Terpenoid backbone biosynthesis
*PER2*	1.58 × 10^−24^	2.74 × 10^0^	3.16 × 10^−5^	0.026	Acute myeloid leukemia, Circadian rhythm-fly
*TLR2A*	5.64 × 10^−5^	9.32 × 10^−1^	1.09 × 10^−3^	0.011	Herpes simplex virus 1 infection, Toll-like receptor signaling pathway
*TLR1A*	1.23 × 10^−2^	6.93 × 10^−1^	1.67 × 10^−3^	0.021	Toll-like receptor signaling pathway, Salmonella infection
*DHCR24*	1.98 × 10^−6^	−2.01 × 10^0^	4.42 × 10^−4^	0.043	Steroid biosynthesis, Metabolic pathways
*SQLE*	6.14 × 10^−9^	−2.42 × 10^0^	3.70 × 10^−3^	0.019	Steroid biosynthesis, Metabolic pathways
*NSDHL*	1.50 × 10^−8^	−2.60 × 10^0^	1.10 × 10^−2^	0.047	Steroid biosynthesis, Metabolic pathways
*IARS1*	1.13 × 10^−5^	−1.22 × 10^0^	2.14 × 10^−2^	0.012	Aminoacyl-tRNA biosynthesis
*ASNS*	6.31 × 10^−7^	−1.54 × 10^0^	2.92 × 10^−2^	0.030	Alanine, aspartate and glutamate metabolism, etc.
*ELOVL2*	2.56 × 10^−14^	−1.92 × 10^0^	2.92 × 10^−2^	0.037	Metabolic pathways, Fatty acid elongation, etc.
*IVD*	2.62 × 10^−2^	−9.45 × 10^−1^	2.92 × 10^−2^	0.035	Metabolic pathways, Valine, leucine and isoleucine degradation
*DHCR7*	2.90 × 10^−6^	−1.84 × 10^0^	3.96 × 10^−2^	0.031	Steroid biosynthesis, Metabolic pathways
*SRSF4*	6.06 × 10^−3^	5.95 × 10^−1^	3.70 × 10^−3^	0.050	Spliceosome
*SRSF5*	1.53 × 10^−2^	7.04 × 10^−1^	3.70 × 10^−3^	0.068	Spliceosome

Note: *p* Value_DEG represents the *p*-value of Differentially Expressed Genes; *p* Value_JTK is the *p*-value of the JTK-Cycle Algorithm; *p* Value_Cosinor is the *p*-value of Cosinor Analysis.

## Data Availability

All data is included in this paper.

## Supplementary Materials

The following supporting information can be downloaded at https://www.mdpi.com/article/10.3390/ani15223241/s1, Figure S1: Soft threshold determination plot under scale-free distribution (β = 14). Figure S2: Variation trends of Profiles 0, 1, 3, and 4. Figure S3: Enriched genes related to circadian rhythm and immunometabolism. Figure S4: Network diagram, word cloud, and KEGG analysis results of all genes in the cyan module. Figure S5: Network diagram, word cloud, and KEGG analysis results of all genes in the tan module. Figure S6: KEGG classes and pathways based on all genes under light conditions. Figure S7: KEGG classes and pathways based on all genes under dark conditions. Figure S8: correlation heatmap of 20 circadian rhythm-differential genes. Table S1: Composition and nutrient levels of experimental diets (air-dry basis, %). Table S2: Primer sequence used for RT−PCR. Table S3: Summary for the number of differentially expressed genes among 6 groups at different time points. Table S4: Differentially expressed genes between the cyan module and the tan module. Table S5: Hub genes of the cyan module and the tan module. Table S6: Protein-coding genes with rhythmic significance.

## References

[1] Meyer N., Harvey A.G., Lockley S.W., Dijk D.J. (2022). Circadian rhythms and disorders of the timing of sleep. Lancet.

[2] King D.P., Takahashi J.S. (2000). Molecular genetics of circadian rhythms in mammals. Annu. Rev. Neurosci..

[3] Begemann K., Neumann A.M., Oster H. (2020). Regulation and function of extra-SCN circadian oscillators in the brain. Acta Physiol..

[4] Zhang R., Lahens N.F., Ballance H.I., Hughes M.E., Hogenesch J.B. (2014). A circadian gene expression atlas in mammals: Implications for biology and medicine. Proc. Natl. Acad. Sci. USA.

[5] Mohawk J.A., Green C.B., Takahashi J.S. (2012). Central and peripheral circadian clocks in mammals. Annu. Rev. Neurosci..

[6] Turek F.W., Joshu C., Kohsaka A., Lin E., Ivanova G., McDearmon E., Laposky A., Losee-Olson S., Easton A., Jensen D.R. (2005). Obesity and metabolic syndrome in circadian Clock mutant mice. Science.

[7] Pan X., Bradfield C.A., Hussain M.M. (2016). Global and hepatocyte-specific ablation of Bmal1 induces hyperlipidaemia and enhances atherosclerosis. Nat. Commun..

[8] Ge W., Sun Q., Yang Y., Ding Z., Liu J., Zhang J. (2023). Circadian PER1 controls daily fat absorption with the regulation of PER1-PKA on phosphorylation of bile acid synthetase. J. Lipid Res..

[9] Aiello I., Mul Fedele M.L., Román F., Marpegan L., Caldart C., Chiesa J.J., Golombek D.A., Finkielstein C.V., Paladino N. (2020). Circadian disruption promotes tumor-immune microenvironment remodeling favoring tumor cell proliferation. Sci. Adv..

[10] Liu Z., Zhang J., Li S., Wang H., Ren B., Li J., Bao Z., Liu J., Guo M., Yang G. (2023). Circadian control of ConA-induced acute liver injury and inflammatory response via Bmal1 regulation of Junb. JHEP Rep..

[11] Narasimamurthy R., Hatori M., Nayak S.K., Liu F., Panda S., Verma I.M. (2012). Circadian clock protein cryptochrome regulates the expression of proinflammatory cytokines. Proc. Natl. Acad. Sci. USA.

[12] Zhang W., Xu X., Zhang R., Tian Y., Ma X., Wang X., Jiang Y., Man C. (2024). Stress-Induced Immunosuppression Inhibits Regional Immune Responses in Chicken Adipose Tissue Partially through Suppressing T Cells by Up-Regulating Steroid Metabolism. Animals.

[13] Farhat A., Buick J.K., Williams A., Yauk C.L., O’Brien J.M., Crump D., Williams K.L., Chiu S., Kennedy S.W. (2014). Tris(1,3-dichloro-2-propyl) phosphate perturbs the expression of genes involved in immune response and lipid and steroid metabolism in chicken embryos. Toxicol. Appl. Pharmacol..

[14] Sun W., Li P., Cai J., Ma J., Zhang X., Song Y., Liu Y. (2022). Lipid Metabolism: Immune Regulation and Therapeutic Prospectives in Systemic Lupus Erythematosus. Front. Immunol..

[15] Zhao H., Wu M., Tang X., Li Q., Yi X., Zhao W., Sun X. (2022). RNA-seq Based Transcriptome Analysis Reveals The Cross-Talk of Macrophage and Adipocyte of Chicken Subcutaneous Adipose Tissue during The Embryonic and Post-Hatch Period. Front. Immunol..

[16] Daniels L.J., Kay D., Marjot T., Hodson L., Ray D.W. (2023). Circadian regulation of liver metabolism: Experimental approaches in human, rodent, and cellular models. Am. J. Physiol. Cell Physiol..

[17] Pati P., Valcin J.A., Zhang D., Neder T.H., Millender-Swain T., Allan J.M., Sedaka R., Jin C., Becker B.K., Pollock D.M. (2021). Liver circadian clock disruption alters perivascular adipose tissue gene expression and aortic function in mice. Am. J. Physiol. Regul. Integr. Comp. Physiol..

[18] Gooley J.J., Chua E.C. (2014). Diurnal regulation of lipid metabolism and applications of circadian lipidomics. J. Genet. Genom..

[19] Frazier K., Manzoor S., Carroll K., DeLeon O., Miyoshi S., Miyoshi J., St George M., Tan A., Chrisler E.A., Izumo M. (2023). Gut microbes and the liver circadian clock partition glucose and lipid metabolism. J. Clin. Investig..

[20] He C., Shen W., Chen C., Wang Q., Lu Q., Shao W., Jiang Z., Hu H. (2021). Circadian Rhythm Disruption Influenced Hepatic Lipid Metabolism, Gut Microbiota and Promoted Cholesterol Gallstone Formation in Mice. Front. Endocrinol..

[21] Wang P., Li F., Sun Y., Li Y., Xie X., Du X., Liu L., Wu Y., Song D., Xiong H. (2024). Novel insights into the circadian modulation of lipid metabolism in chicken livers revealed by RNA sequencing and weighted gene co-expression network analysis. Poult. Sci..

[22] Selvaraj S., Oh J.H., Yoon S., Borlak J. (2023). Diclofenac Disrupts the Circadian Clock and through Complex Cross-Talks Aggravates Immune-Mediated Liver Injury-A Repeated Dose Study in Minipigs for 28 Days. Int. J. Mol. Sci..

[23] Chen S. (2023). Ultrafast one-pass FASTQ data preprocessing, quality control, and deduplication using fastp. Imeta.

[24] Brown J., Pirrung M., McCue L.A. (2017). FQC Dashboard: Integrates FastQC results into a web-based, interactive, and extensible FASTQ quality control tool. Bioinformatics.

[25] Thakur V. (2024). RNA-Seq Data Analysis for Differential Gene Expression Using HISAT2-StringTie-Ballgown Pipeline. Methods Mol. Biol..

[26] Danecek P., Bonfield J.K., Liddle J., Marshall J., Ohan V., Pollard M.O., Whitwham A., Keane T., McCarthy S.A., Davies R.M. (2021). Twelve years of SAMtools and BCFtools. Gigascience.

[27] Pertea M., Kim D., Pertea G.M., Leek J.T., Salzberg S.L. (2016). Transcript-level expression analysis of RNA-seq experiments with HISAT, StringTie and Ballgown. Nat. Protoc..

[28] Love M.I., Huber W., Anders S. (2014). Moderated estimation of fold change and dispersion for RNA-seq data with DESeq2. Genome Biol..

[29] Langfelder P., Horvath S. (2008). WGCNA: An R package for weighted correlation network analysis. BMC Bioinform..

[30] Wu G., Anafi R.C., Hughes M.E., Kornacker K., Hogenesch J.B. (2016). MetaCycle: An integrated R package to evaluate periodicity in large scale data. Bioinformatics.

[31] Rychlik W. (2007). OLIGO 7 primer analysis software. Methods Mol. Biol..

[32] Yahoo N., Dudek M., Knolle P., Heikenwälder M. (2023). Role of immune responses in the development of NAFLD-associated liver cancer and prospects for therapeutic modulation. J. Hepatol..

[33] Zhou X., Wan D., Zhang Y., Zhang Y., Long C., Chen S., He L., Tan B., Wu X., Yin Y. (2017). Diurnal variations in polyunsaturated fatty acid contents and expression of genes involved in their de novo synthesis in pigs. Biochem. Biophys. Res. Commun..

[34] Acimovic J., Fink M., Pompon D., Bjorkhem I., Hirayama J., Sassone-Corsi P., Golicnik M., Rozman D. (2008). CREM modulates the circadian expression of CYP51, HMGCR and cholesterogenesis in the liver. Biochem. Biophys. Res. Commun..

[35] Tessier L., Côté O., Clark M.E., Viel L., Diaz-Méndez A., Anders S., Bienzle D. (2017). Impaired response of the bronchial epithelium to inflammation characterizes severe equine asthma. BMC Genom..

[36] Masri S., Sassone-Corsi P. (2018). The emerging link between cancer, metabolism, and circadian rhythms. Nat. Med..

[37] Lu Y., Huang D., Wang B., Zheng B., Liu J., Song J., Zheng S. (2021). FAM21C Promotes Hepatocellular Carcinoma Invasion and Metastasis by Driving Actin Cytoskeleton Remodeling via Inhibiting Capping Ability of CAPZA1. Front. Oncol..

[38] Lee K.M., Wu C.C., Fan Y.T., Chiang H.J., Lien P.Y., Wang J.P., Huang Y.C., Shih S.R. (2025). Subversion of phosphorylated SR proteins by enterovirus A71 in IRES-dependent translation revealed by RNA-interactome analysis. PLoS Pathog..

[39] Kim H.J., Jeong E.K., Lee H.J., Jung Y.J. (2025). The photosensitizer DH-I-180-3 regulates intracellular bacterial growth by increasing the secretion of proinflammatory cytokines via the NF-κB- and MAPK-mediated signaling pathways and promoting phagosome maturation in Salmonella-infected mouse macrophages. J. Microbiol..

[40] Li Z., Wu H., Fu J., Mushtaq M., Khan M., Liu Y., Azeem Z., Shi H., He Y., Zhang R. (2024). Eggshell Quality Traits and Transcriptome Gene Screening Between Yunnong and Jingfen Chicken Breeds. Biology.

[41] Jia Q., Cao Y., Zhang M., Xing Y., Xia T., Guo Y., Yue Y., Li X., Liu X., Zhang Y. (2024). miR-19b-3p regulated by estrogen controls lipid synthesis through targeting MSMO1 and ELOVL5 in LMH cells. Poult. Sci..

[42] Liu L., Liu X., Cui H., Liu R., Zhao G., Wen J. (2019). Transcriptional insights into key genes and pathways controlling muscle lipid metabolism in broiler chickens. BMC Genom..

[43] Zhang S., Fang X., Wang Z., Bordbar F., Lin J., Liu M., Li Z. (2025). VNN2 regulates hepatic steroid synthesis in response to dietary changes. Gene.

[44] Gregory M.K., Cleland L.G., James M.J. (2013). Molecular basis for differential elongation of omega-3 docosapentaenoic acid by the rat Elovl5 and Elovl2. J. Lipid Res..

[45] Durna Ö., Hitit M., Usta Z., Yildiz G. (2025). Changes in the Expression of Some Genes With Metabolic, VLDL and Antioxidative Effects After the Addition of Essential Oil Mixture to Drinking Water in the Liver of Domestic Geese (Anser anser Domesticus). Vet. Med. Sci..

[46] Tang J., Fang Q., Shao R., Shen J., He J., Niu D., Lu L. (2018). Digital gene-expression profiling analysis of the fatty liver of Landes geese fed different supplemental oils. Gene.

[47] Cánovas A., Quintanilla R., Gallardo D., Díaz I., Noguera J.L., Ramírez O., Pena R.N. (2010). Functional and association studies on the pig HMGCR gene, a cholesterol-synthesis limiting enzyme. Animal.

[48] Afonso M.S., Machado R.M., Lavrador M.S., Quintao E.C.R., Moore K.J., Lottenberg A.M. (2018). Molecular Pathways Underlying Cholesterol Homeostasis. Nutrients.

[49] Wang H., Wu K., Mi X., Rajput S.A., Qi D. (2023). Effects of 3-Hydroxy-3-methylglutaryl-CoA Reductase Inhibitors on Cholesterol Metabolism in Laying Hens. Animals.

[50] Du X., Ren J.D., Xu X.Q., Chen G.H., Huang Y., Du J.P., Tao Z.R., Cai Z.X., Lu L.Z., Yang H. (2019). Comparative transcriptome analysis reveals genes related to the yolk ratio of duck eggs. Anim. Genet..

[51] Rao S.W., Duan Y.Y., Zhao D.S., Liu C.J., Xu S.H., Liang D., Zhang F.X., Shi W. (2022). Integrative Analysis of Transcriptomic and Metabolomic Data for Identification of Pathways Related to Matrine-Induced Hepatotoxicity. Chem. Res. Toxicol..

[52] Guan D., Lazar M.A. (2022). Circadian Regulation of Gene Expression and Metabolism in the Liver. Semin. Liver Dis..

[53] Zhou C., Hu Z., Liu X., Wang Y., Wei S., Liu Z. (2024). Disruption of the peripheral biological clock may play a role in sleep deprivation-induced dysregulation of lipid metabolism in both the daytime and nighttime phases. Biochim. Biophys. Acta Mol. Cell Biol. Lipids.

[54] Paredes J.F., López-Olmeda J.F., Martínez F.J., Sánchez-Vázquez F.J. (2015). Daily rhythms of lipid metabolic gene expression in zebra fish liver: Response to light/dark and feeding cycles. Chronobiol. Int..

[55] Feng C., Sarigaiqiqige, Liu W., Chen H., Dong W., Yang J. (2021). Effect of dark environment on intestinal flora and expression of genes related to liver metabolism in zebrafish (Danio rerio). Comp. Biochem. Physiol. C Toxicol. Pharmacol..

[56] Petrenko V., Sinturel F., Riezman H., Dibner C. (2023). Lipid metabolism around the body clocks. Prog. Lipid Res..

[57] Zhen Y., Wang Y., He F., Chen Y., Hu L., Ge L., Wang Y., Wei W., Rahmat A., Loor J.J. (2023). Homeostatic crosstalk among gut microbiome, hypothalamic and hepatic circadian clock oscillations, immunity and metabolism in response to different light-dark cycles: A multiomics study. J. Pineal Res..

[58] Zhang B., Yu C., Xu Y., Huang Z., Cai Y., Li Y. (2023). Hepatopancreas immune response during different photoperiods in the Chinese mitten crab, Eriocheir sinensis. Fish. Shellfish. Immunol..

[59] Scanes C.G., Campbell R., Griminger P. (1987). Control of energy balance during egg production in the laying hen. J. Nutr..

[60] Dauchy R.T., Sauer L.A., Blask D.E., Vaughan G.M. (1997). Light contamination during the dark phase in “photoperiodically controlled” animal rooms: Effect on tumor growth and metabolism in rats. Lab. Anim. Sci..

[61] Cissé Y.M., Russart K., Nelson R.J. (2020). Exposure to dim light at night prior to conception attenuates offspring innate immune responses. PLoS ONE.

[62] Cissé Y.M., Russart K.L., Nelson R.J. (2017). Parental Exposure to Dim Light at Night Prior to Mating Alters Offspring Adaptive Immunity. Sci. Rep..

[63] Ma J., Abram C.L., Hu Y., Lowell C.A. (2019). CARD9 mediates dendritic cell-induced development of Lyn deficiency-associated autoimmune and inflammatory diseases. Sci. Signal.

[64] Buniyaadi A., Prabhat A., Bhardwaj S.K., Kumar V. (2025). Role of melatonin in physiological mitigation of sleep disruption in an unnatural temporal environment. J. Neuroendocrinol..

[65] Yang Y., Yang L., Qi C., Hu G., Wang L., Sun Z., Ni X. (2020). Cryptotanshinone alleviates polycystic ovary syndrome in rats by regulating the HMGB1/TLR4/NF-κB signaling pathway. Mol. Med. Rep..

[66] Lu Z., Li X., Wang M., Zhang X., Zhuang R., Wu F., Li W., Zhu W., Zhang B. (2023). Liver-Specific Bmal1 Depletion Reverses the Beneficial Effects of Nobiletin on Liver Cholesterol Homeostasis in Mice Fed with High-Fat Diet. Nutrients.

