# Supplementary material for: Transcriptome Analysis Reveals Circadian Rhythmic Regulation of Lipid Metabolism and Immune Function in Chicken Livers
Source: Animals (Basel). 2025 Nov 8;15(22):3241. doi: 10.3390/ani15223241 (PMC12649623; doi:10.3390/ani15223241)
Supplement: Supplementary file 1 [file animals-15-03241-s001.zip › Figure S5.pdf]

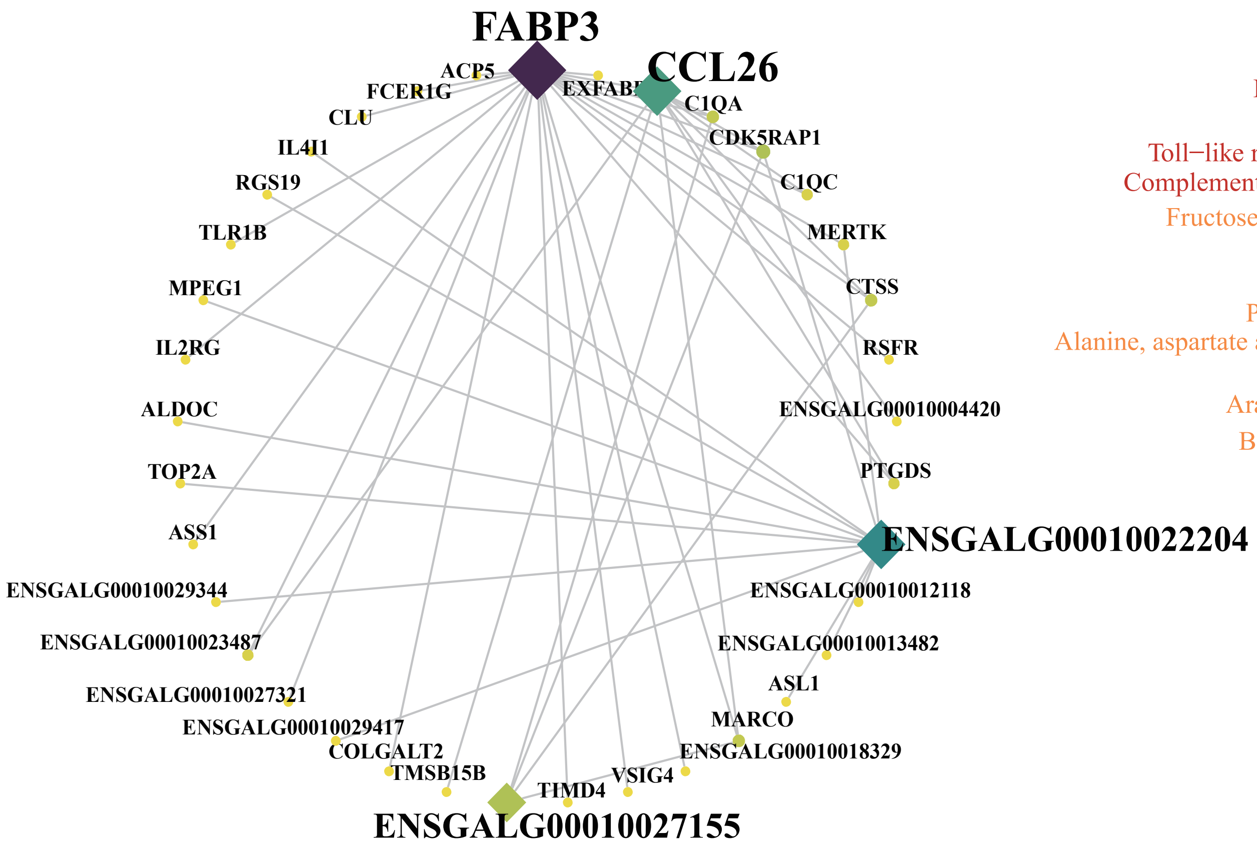
FABP3
CCL26
ACP5
FCER1G
EXFABP
C1QA
CLU
CDK5RAP1
IL4I1
C1QC
RGS19
TLR1B
MERTK
MPEG1
CTSS
IL2RG
RSFR
ALDOC
ENSGALG00010004420
TOP2A
PTGDS
ASS1
ENSGALG00010022204
ENSGALG00010029344
ENSGALG00010012118
ENSGALG00010023487
ENSGALG00010013482
ENSGALG00010027321
ASL1
ENSGALG00010029417
MARCO
COLGALT2
ENSGALG00010018329
TMSB15B
VSIG4
TIMD4
ENSGALG00010027155

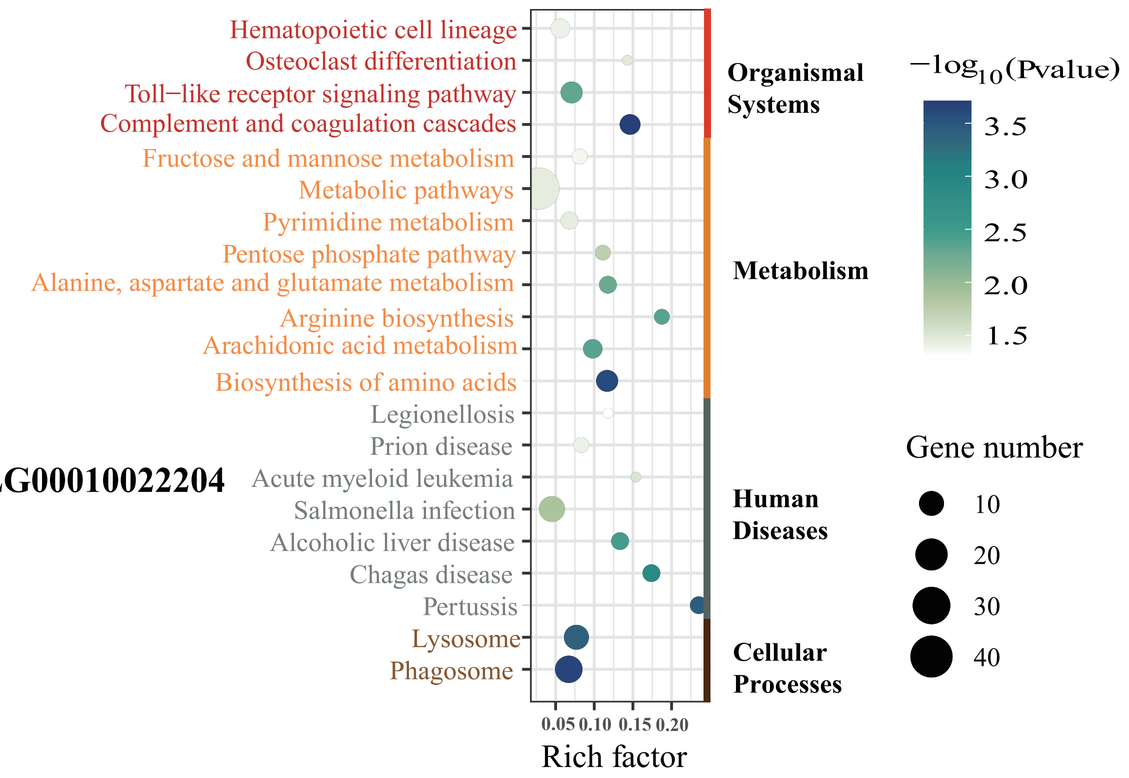
Hematopoietic cell lineage
Osteoclast differentiation
Toll−like receptor signaling pathway
Complement and coagulation cascades
Fructose and mannose metabolism
Metabolic pathways
Pyrimidine metabolism
Pentose phosphate pathway
Alanine, aspartate and glutamate metabolism
Arginine biosynthesis
Arachidonic acid metabolism
Biosynthesis of amino acids
Legionellosis
Prion disease
Acute myeloid leukemia
Salmonella infection
Alcoholic liver disease
Chagas disease
Pertussis
Lysosome
Phagosome
0.05 0.10 0.15 0.20
Rich factor
Organismal Systems
Metabolism
Human Diseases
Cellular Processes
$-\log_{10}$(Pvalue)
3.5
3.0
2.5
2.0
1.5
Gene number
10
20
30
40

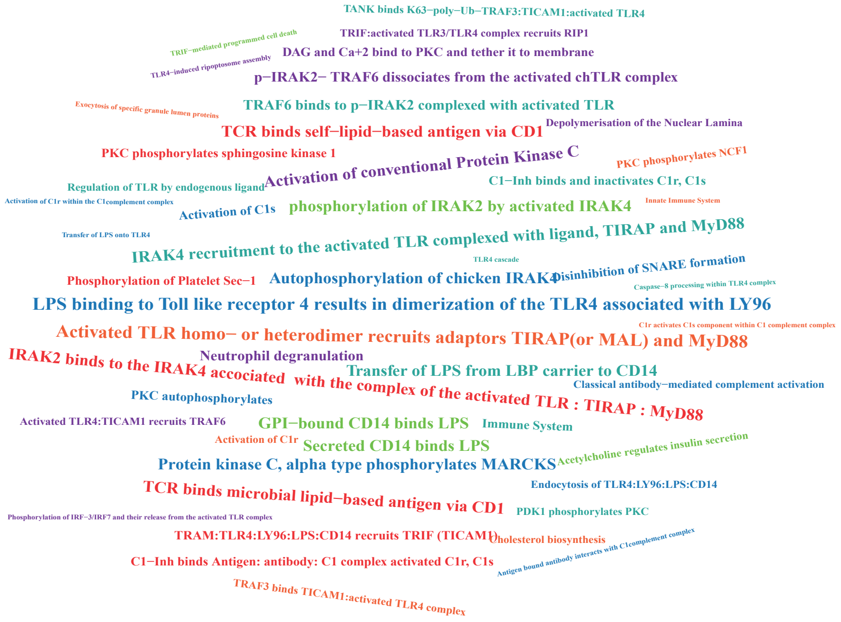
TANK binds K63−poly−Ub−TRAF3:TICAM1:activated TLR4
TRIF:activated TLR3/TLR4 complex recruits RIP1
TRIF−mediated programmed cell death
DAG and Ca+2 bind to PKC and tether it to membrane
TLR4−induced ripoptosome assembly
p−IRAK2− TRAF6 dissociates from the activated chTLR complex
Exocytosis of specific granule lumen proteins
TRAF6 binds to p−IRAK2 complexed with activated TLR
TCR binds self−lipid−based antigen via CD1
Depolymerisation of the Nuclear Lamina
PKC phosphorylates sphingosine kinase 1
Activation of conventional Protein Kinase C
PKC phosphorylates NCF1
Regulation of TLR by endogenous ligand
C1−Inh binds and inactivates C1r, C1s
Activation of C1r within the C1complement complex
Activation of C1s
phosphorylation of IRAK2 by activated IRAK4
Innate Immune System
Transfer of LPS onto TLR4
IRAK4 recruitment to the activated TLR complexed with ligand, TIRAP and MyD88
TLR4 cascade
Phosphorylation of Platelet Sec−1
Autophosphorylation of chicken IRAK4
Disinhibition of SNARE formation
Caspase−8 processing within TLR4 complex
LPS binding to Toll like receptor 4 results in dimerization of the TLR4 associated with LY96
Activated TLR homo− or heterodimer recruits adaptors TIRAP(or MAL) and MyD88
C1r activates C1s component within C1 complement complex
IRAK2 binds to the IRAK4 accociated with the complex of the activated TLR : TIRAP : MyD88
Neutrophil degranulation
Transfer of LPS from LBP carrier to CD14
Classical antibody−mediated complement activation
PKC autophosphorylates
Activated TLR4:TICAM1 recruits TRAF6
GPI−bound CD14 binds LPS
Immune System
Activation of C1r
Secreted CD14 binds LPS
Protein kinase C, alpha type phosphorylates MARCKS
Acetylcholine regulates insulin secretion
Endocytosis of TLR4:LY96:LPS:CD14
TCR binds microbial lipid−based antigen via CD1
PDK1 phosphorylates PKC
Phosphorylation of IRF−3/IRF7 and their release from the activated TLR complex
TRAM:TLR4:LY96:LPS:CD14 recruits TRIF (TICAM1)
Cholesterol biosynthesis
Antigen bound antibody interacts with C1complement complex
C1−Inh binds Antigen: antibody: C1 complex activated C1r, C1s
TRAF3 binds TICAM1:activated TLR4 complex
