# Supplementary figures and images for: Transcriptome Analysis Reveals Circadian Rhythmic Regulation of Lipid Metabolism and Immune Function in Chicken Livers
Source: Animals (Basel). 2025 Nov 8;15(22):3241. doi: 10.3390/ani15223241 (PMC12649623; doi:10.3390/ani15223241)

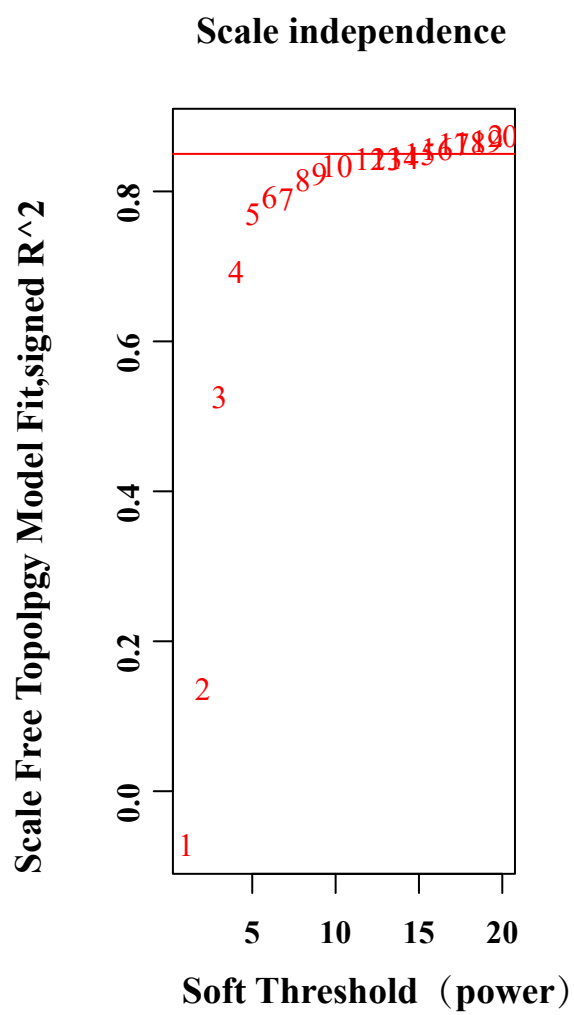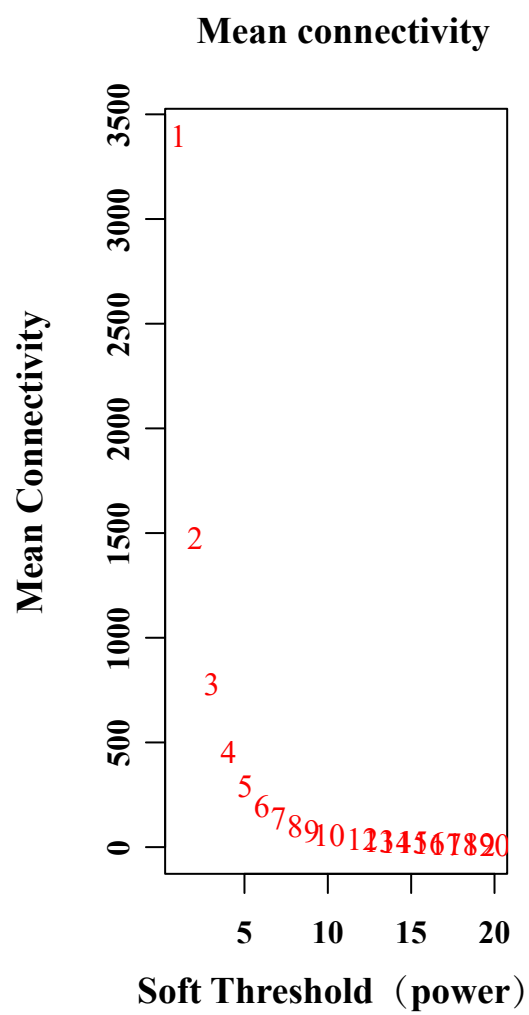

Supplement: Supplementary file 1 [file animals-15-03241-s001.zip › Figure S1.pdf]

**Profile 0 : 589 Genes**

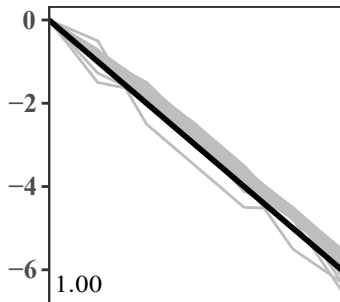

**Profile 1 : 627 Genes**

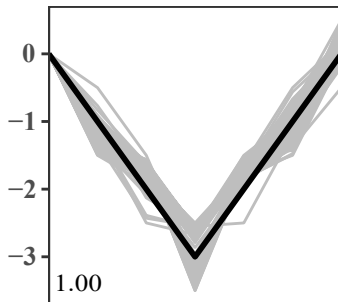

**Profile 3 : 708 Genes**

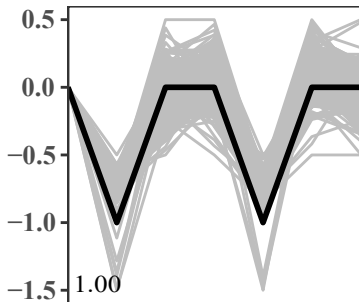

**Profile 4 : 189 Genes**

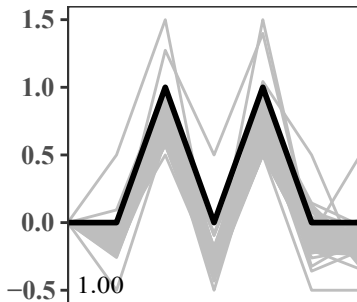

Supplement: Supplementary file 1 [file animals-15-03241-s001.zip › Figure S2.pdf]

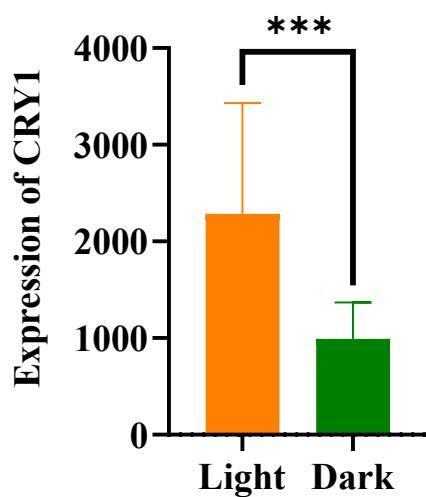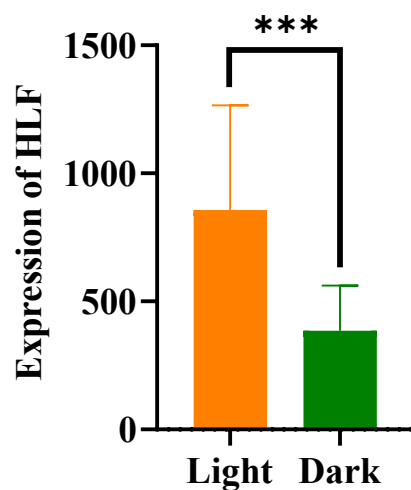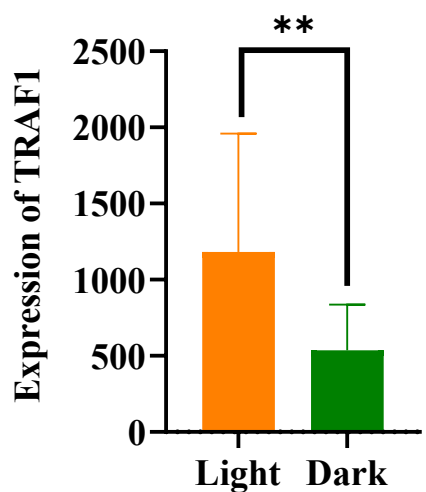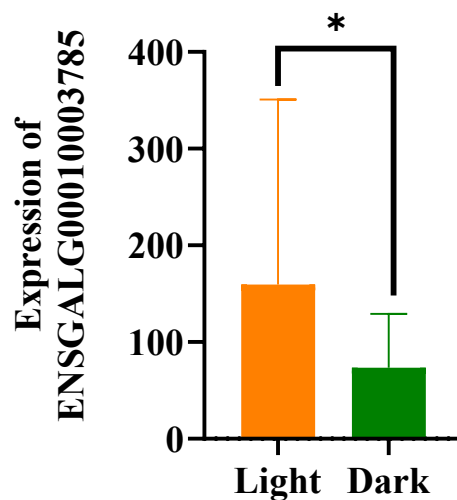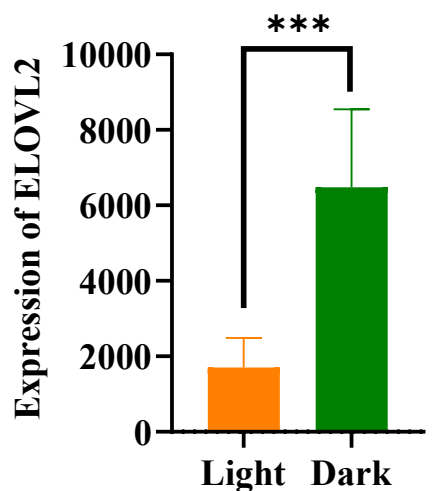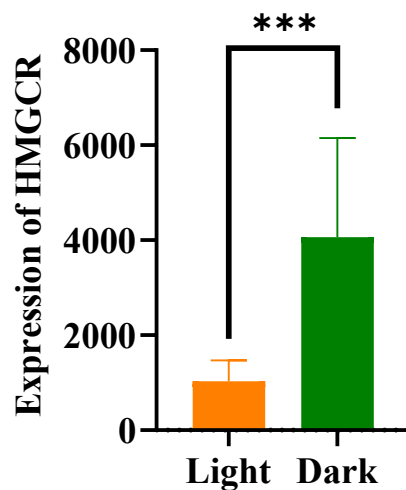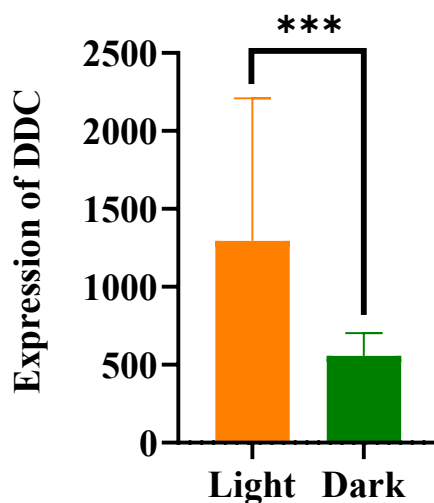

Supplement: Supplementary file 1 [file animals-15-03241-s001.zip › Figure S3.pdf]

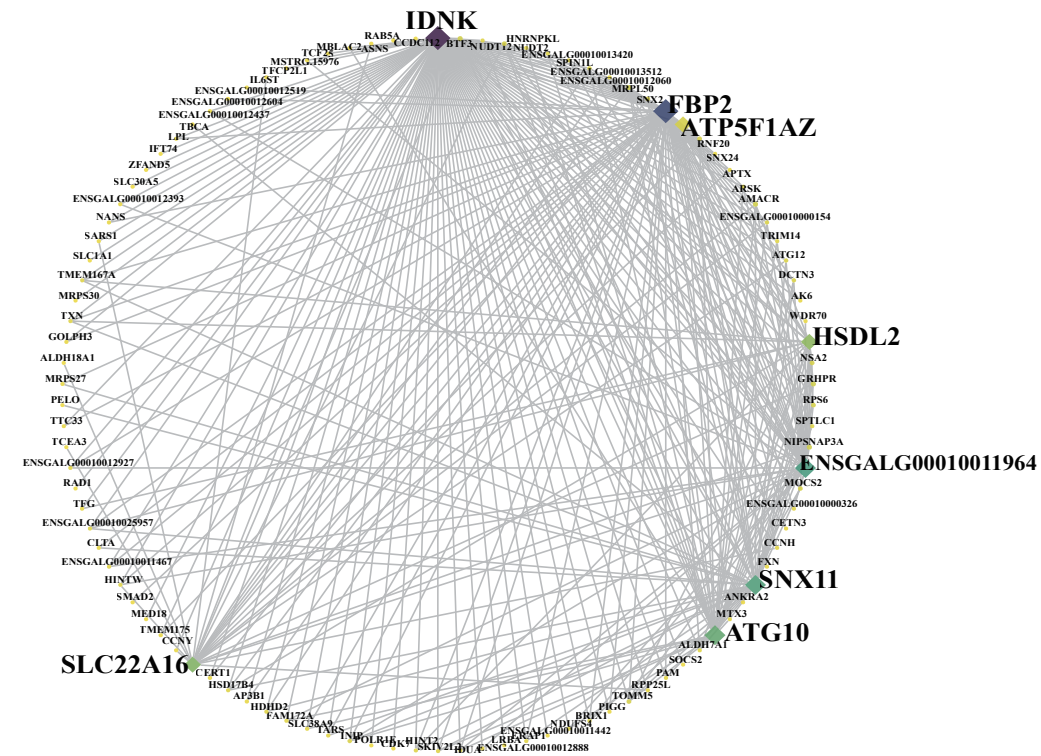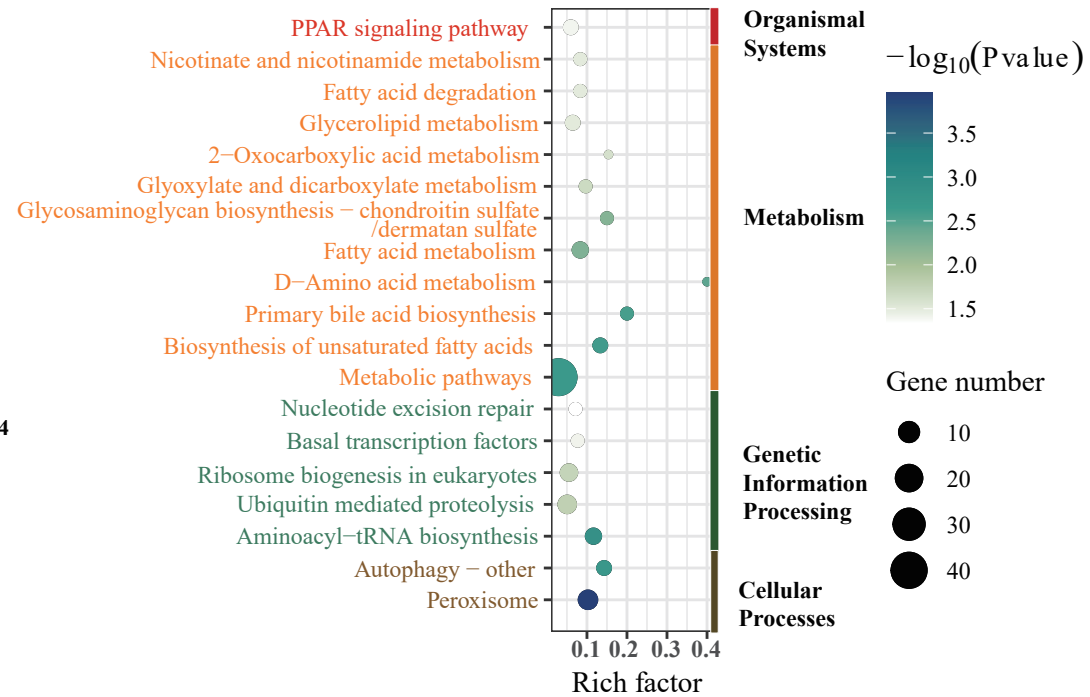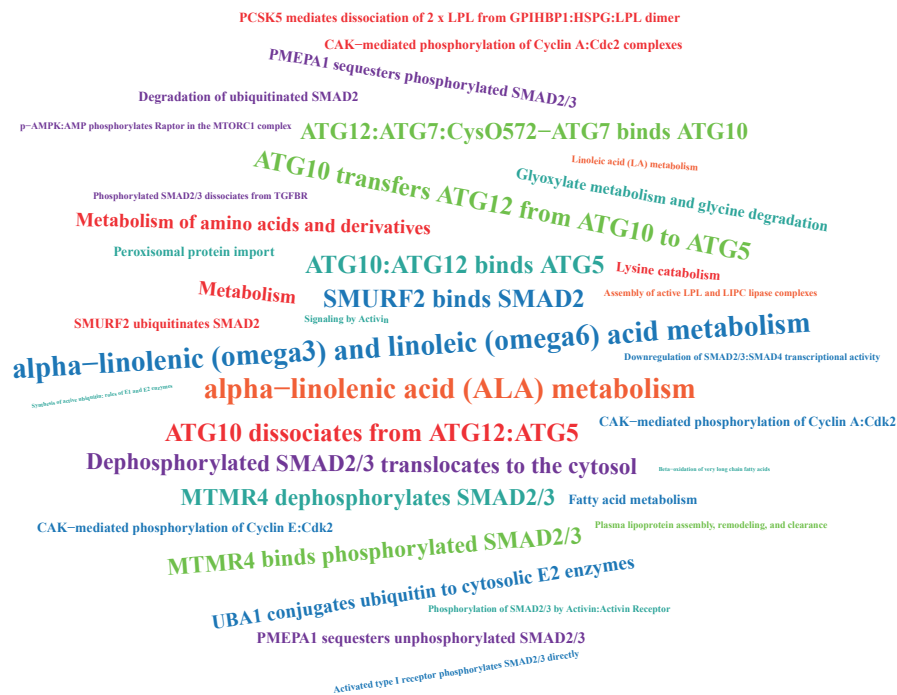

Supplement: Supplementary file 1 [file animals-15-03241-s001.zip › Figure S4.pdf]

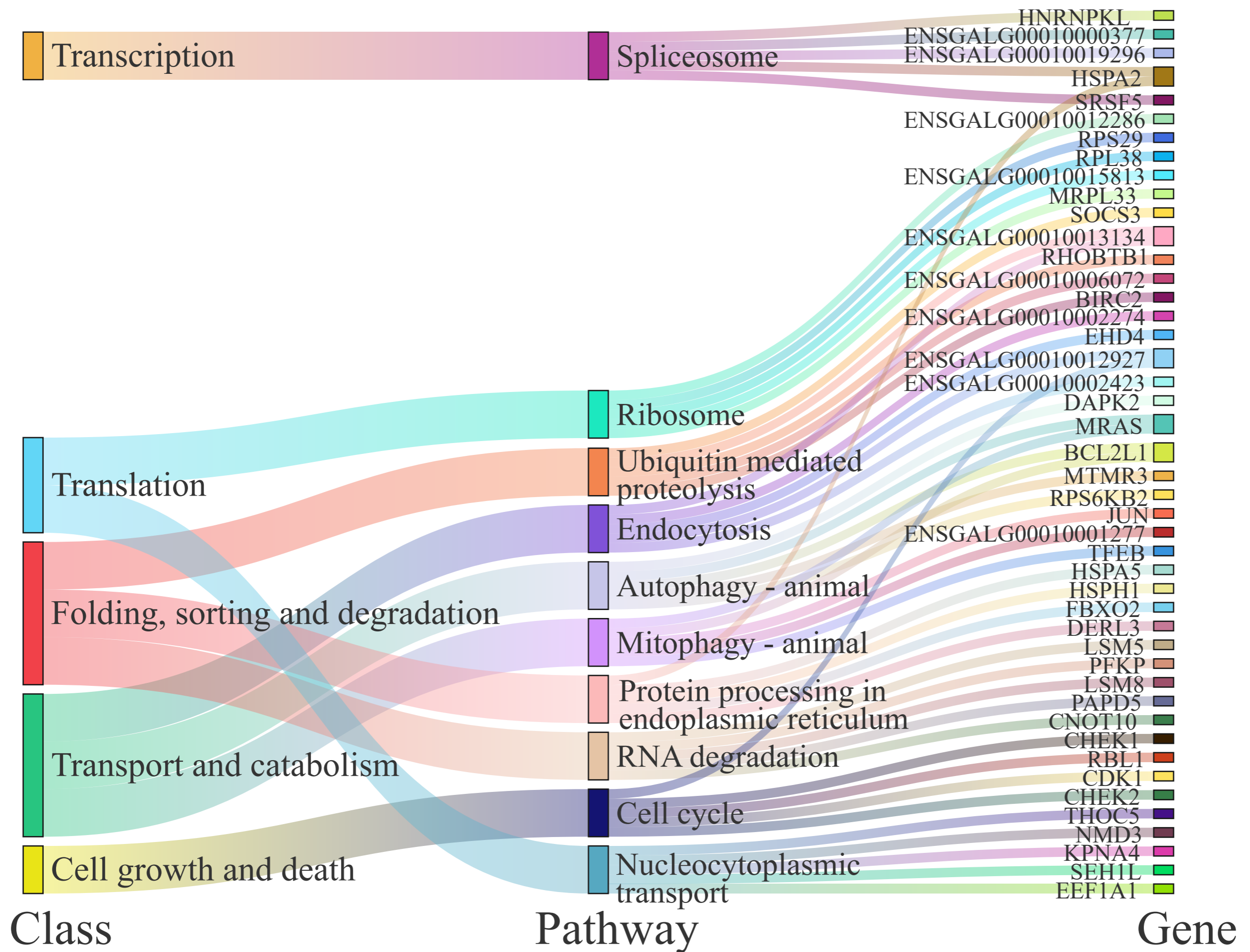

Supplement: Supplementary file 1 [file animals-15-03241-s001.zip › Figure S6.pdf]

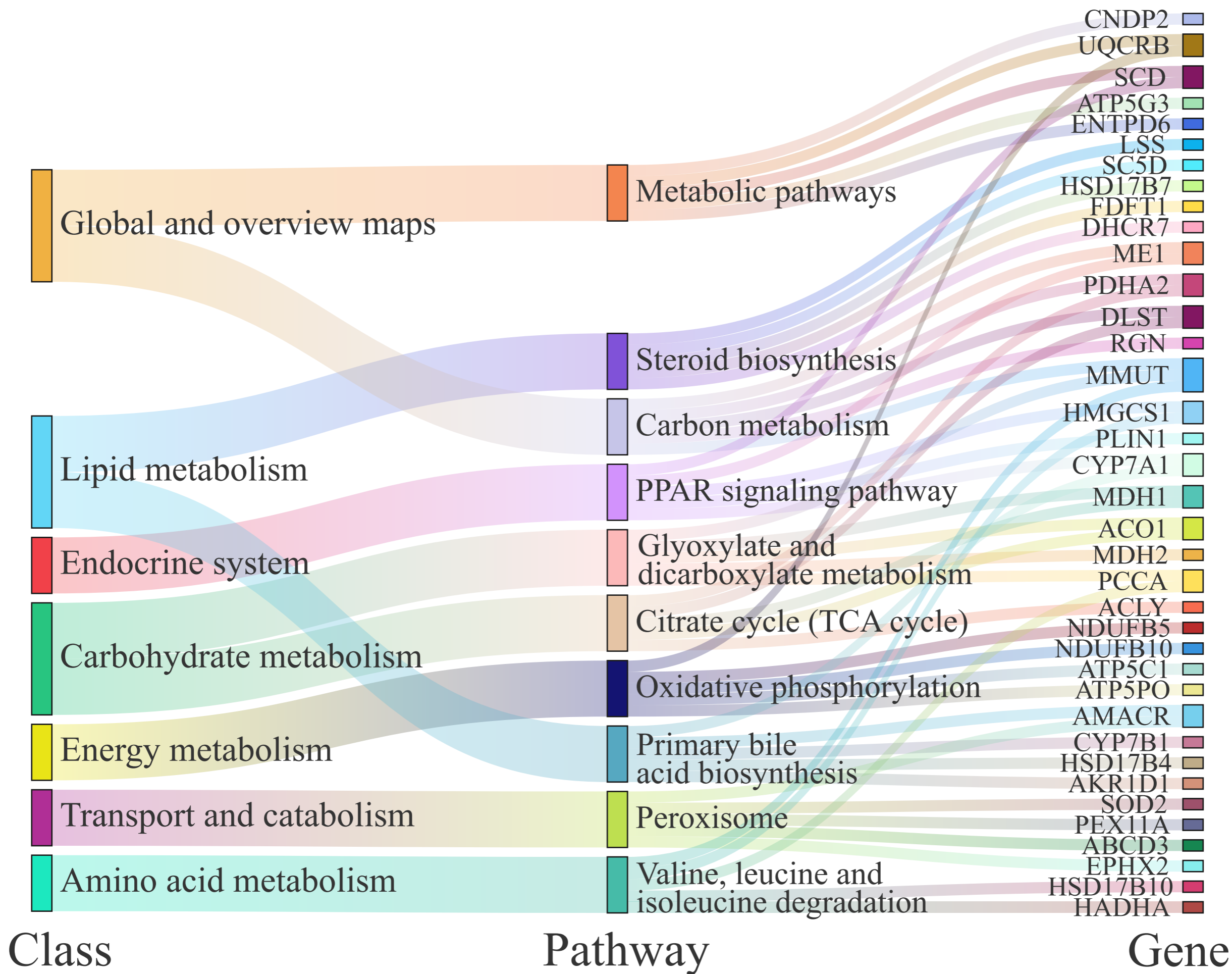

Supplement: Supplementary file 1 [file animals-15-03241-s001.zip › Figure S7.pdf]

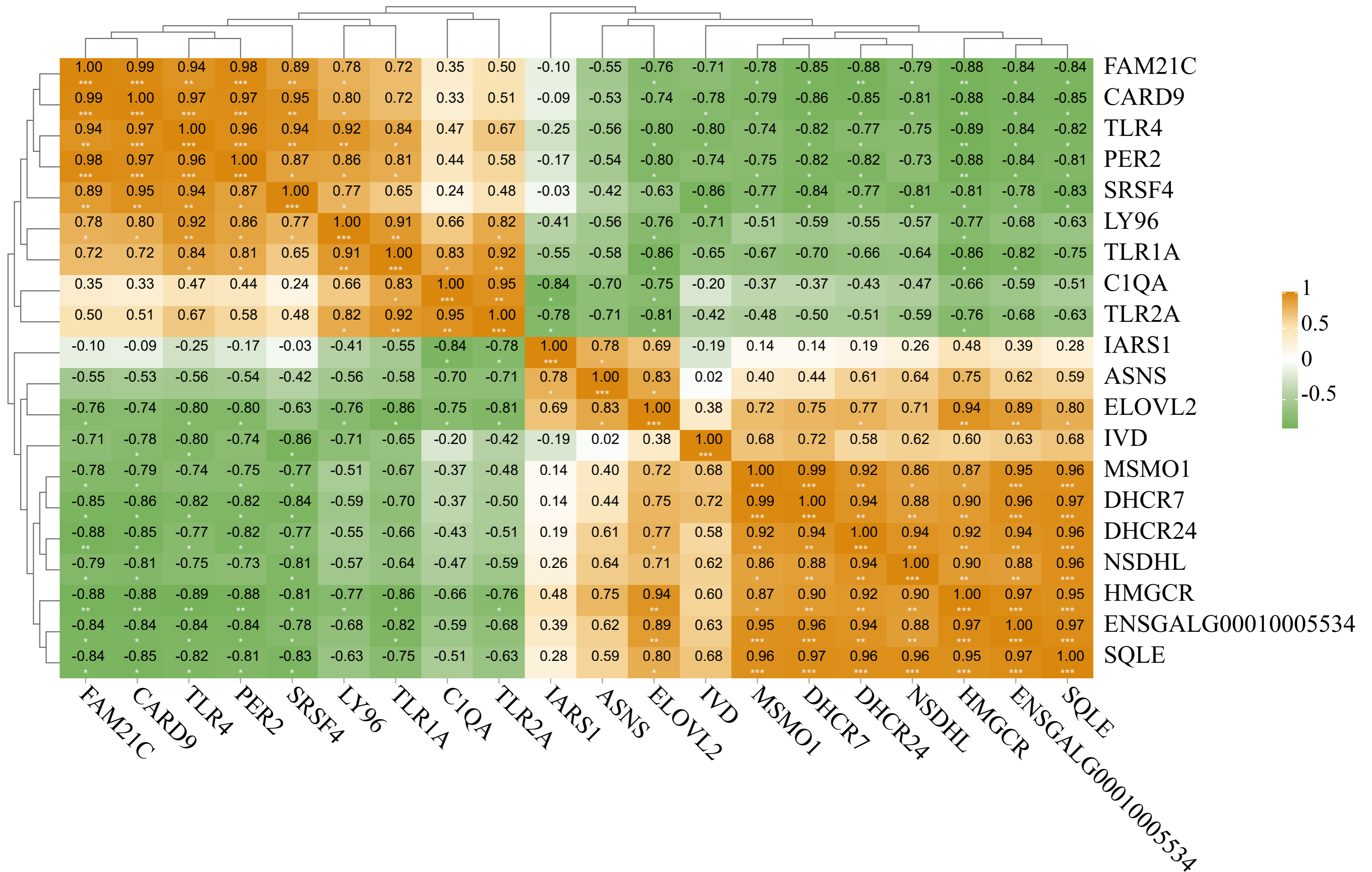

Supplement: Supplementary file 1 [file animals-15-03241-s001.zip › Figure S8.pdf]
